# Ultrasound-Guided Venous Puncture Reduces Groin Complications in Electrophysiological Procedures

**DOI:** 10.3390/biomedicines12102375

**Published:** 2024-10-17

**Authors:** Yannick Teumer, Daniel Eckart, Lyuboslav Katov, Markus Graf, Carlo Bothner, Wolfgang Rottbauer, Karolina Weinmann-Emhardt

**Affiliations:** 1 Ulm University Heart Center, Ulm University, Albert-Einstein-Allee 23, 89081 Ulm, Germany; yannick.teumer@uniklinik-ulm.de (Y.T.); lyuboslav.katov@uniklinik-ulm.de (L.K.); carlo.bothner@uniklinik-ulm.de (C.B.);; 2 Department of Diagnostic Radiology, Technical University of Munich, Ismaninger Straße 22, 80333 Munich, Germany; markus.m.graf@tum.de

**Keywords:** cardiology, electrophysiology, ultrasound, venipuncture, vascular access complication, bleeding, pseudoaneurysm, arteriovenous fistula

## Abstract

**Background**: In electrophysiological procedures, multiple punctures on the femoral vein can be necessary depending on the number of catheters required. The femoral vein is typically located indirectly by using its anatomical relationship to the artery as a reference. However, this conventional approach can lead to significant complications, including bleeding, peri-interventional transfusion, pseudoaneurysms, or arteriovenous fistulas. Despite these risks, there is limited evidence comparing the safety of ultrasound-guided venipuncture versus the conventional technique in electrophysiological procedures. **Objective**: This study aimed to evaluate the impact of ultrasound-guided venipuncture on vascular access complications in electrophysiological procedures and to identify associated risk factors. Methods: In this single-center trial, patients scheduled for electrophysiological procedures at Ulm University Heart Center, Germany, were enrolled between November 2021 and October 2023. Venipuncture in the groin was performed using either the conventional or an ultrasound-guided approach. The primary composite endpoint was defined as peri-interventional major vascular access complications (Bleeding Academic Research Consortium (BARC) ≥2 bleeding, pseudoaneurysms, arteriovenous fistulas, and peri-interventional transfusion) and minor complications (BARC 1). **Results**: A total of 1370 patients were included: 749 in the conventional group and 621 in the ultrasound group. The primary endpoint was achieved in 19.2% of the conventional group and 12.1% of the ultrasound group (*p* < 0.001). An increased sheath diameter and a higher number of venous accesses were identified as risk factors for the primary endpoint. **Conclusions**: Ultrasound guidance for venous groin puncture in electrophysiological procedures reduces access-related complications, supporting its use with careful attention to sheath size and number.

## 1. Introduction

Unlike other interventions in cardiology, electrophysiological (EP) procedures often require multiple venipunctures in the groin. Given the widespread use of oral anticoagulation in EP patients, they appear to be a high-risk population for access-related complications [1]. In fact, these complications are among the most common issues encountered in patients undergoing atrial fibrillation ablation [2,3], resulting in prolonged hospitalization and costs [4].

The number of venipunctures and the required sheath diameter is determined by the type of tachycardia being treated, the specific workflow, and the technique of the procedure [5]. Unlike arteries, which can be palpated directly, veins in the groin can only be located indirectly by using anatomical knowledge of their typical path relative to the palpable artery. However, significant inter-individual variability in the course of the venous vessels relative to the femoral artery is well documented [6]. As a result, conventional landmark-guided venipuncture carries the risk of severe access-related complications, including significant bleeding, peri-interventional transfusion, pseudoaneurysms, and arteriovenous fistulas. In contrast, ultrasound can be used to visualize the course of the vessel and the puncture needle in real time during the puncture [7,8]. This makes it possible to reduce vascular injury and to avoid accidental arterial punctures [9,10,11].

Despite the known benefits of ultrasound-guided venipuncture, its use is underutilized in EP procedures [2,12,13], possibly due to limited supporting evidence specific to EP populations.

Furthermore, the effects of the number of venous accesses and the sheath diameter used during EP procedures have not been sufficiently investigated. Therefore, the aim of this study was to evaluate the influence of ultrasound-guided venipuncture in the groin compared to conventional landmark-guided puncture in EP procedures. Additionally, the study sought to assess the impact of the number of venous accesses and sheath diameter on the incidence of venous vascular access complications (VACs).

## 2. Materials and Methods

### 2.1. Study Design

We conducted this study between November 2021 and October 2023 at the Ulm University Heart Center, Germany. The inclusion criteria were the patient’s participation in the Ulm Arrhythmia Registry, a scheduled and completed EP procedure at our center during the study period, and being over 18 years of age at the time of the procedure. No exclusion criteria were applied. All procedures were carried out under uninterrupted oral anticoagulation. For venipuncture in the groin, we used anatomical landmarks from November 2021 to October 2022 (conventional group) and switched to ultrasound guidance from November 2022 to October 2023 (ultrasound group). We collected the data as part of the Ulm Arrhythmia Registry (German Clinical Trials Register ID: DRKS00013013). The study complies with the Declaration of Helsinki and was approved by the local ethics committee at Ulm University.

### 2.2. Groin Puncture and Ultrasound Guidance

Experienced interventionalists performed all procedures and venous groin punctures under local anesthesia and sedation depending on the performed procedure. The punctures were performed below the inguinal ligament. A single-wall puncture with an 18 G puncture needle of the femoral vein was always aimed for. The groin punctures were performed on the right side. All punctures for the procedure were performed on the same side. Depending on the procedure to be performed and the number of vascular accesses required, one to a maximum of six venous accesses were performed at the begin of the procedure.

**Figure 1:**
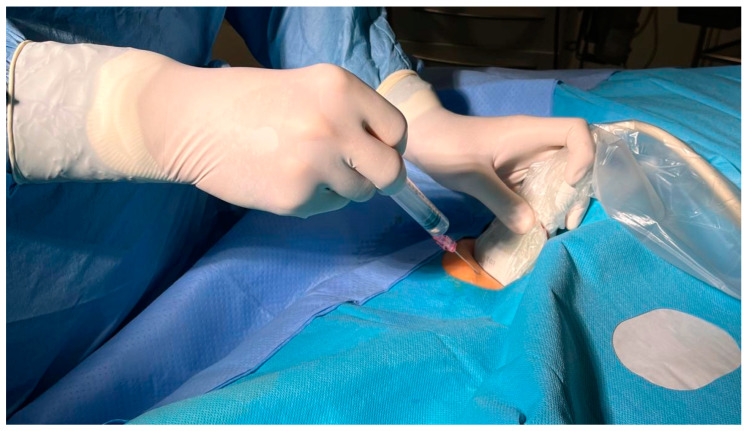
Depiction of ultrasound guidance during venipuncture in the groin during electrophysiological procedures. The illustration shows the puncture of the venous vessels in the groin during an electrophysiological procedure under ultrasound guidance.

**Figure 2:**
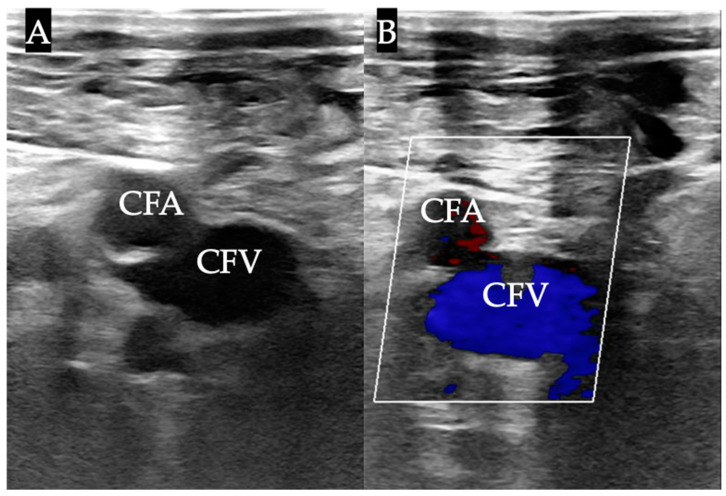
Example of a two-dimensional ultrasound transversal view of the groin vessels with and without color Doppler. Ultrasound images demonstrating a transversal view of the right groin, showing the femoral vessels. The femoral artery and vein are visualized in a transverse plane without (**A**) and with color Doppler ((**B**), artery: dark red color, vein: blue color), providing clear differentiation between the vascular structures. CFA, common femoral artery; CFV, common femoral vein.

In the ultrasound group, the puncture was performed using the out-of-plane technique with a linear transducer (Philips CX50 ultrasound system, in combination with a Philips 12-3 linear ultrasound probe, Philips, Amsterdam, The Netherlands) with a sterile cover (Figure 1). Apart from anatomical knowledge, compression sonography and the color Doppler technique (Figure 2) were used to identify the vein. We inserted the needle at an angle of 30–45 degrees, depending on the depth of the vein. The needle was advanced until blood could be aspirated. After advancing a guidewire through the needle into the vein, we confirmed the correct intraluminal placement of the guidewire using ultrasound.

Various introducer, transseptal, and arterial sheaths of different diameters were used depending on the procedure. Introducer sheaths were used with a diameter between 4 and 8 French (Radifocus Introducer II Standard, Terumo, Japan). Transseptal sheaths with diameters ranging from 8.5 to 13.5 French were used (Vizigo, 8.5F, Biosense Webster, Irwine, CA, USA; Agilis, 8.5F, Abbott, Chicago, IL, USA; GuideStar, 13.5F, Biosense Webster; PolarSheath, 12.7F, Boston Scientific, Marlborough, MA, USA; FlexCath Advance, 12F, Medtronic, Minneapolis, MN, USA).

If necessary, arterial puncture was performed for blood pressure monitoring or retrograde access to the left ventricle. Either an introducer sheath (Radifocus Introducer II Standard, 4F, Terumo, Tokyo, Japan) or a sheathed sheath (Super Arrow-Flex, 8F, 65 cm, Teleflex, Wayne, PA, USA) was used. When performed, the 8F arterial puncture site was closed with a vascular closure device (Angio-Seal, Terumo).

### 2.3. Periprocedural Management

Upon admission, coagulation parameters, including PTT and INR, were routinely measured. During left atrial interventions, a bolus of 10.000 IU heparin was administered, followed by continuous infusion, with a target ACT of 300 to 350 ms. Patients undergoing right atrial ablations received a bolus of 5000 IU heparin prior to ablation, while those undergoing right atrial diagnostic electrophysiological studies did not receive periprocedural anticoagulation with heparin.

At the end of the procedure, the venous sheaths and wires were removed. For procedures involving sheaths up to 7 French, manual compression was applied at the groin until bleeding stopped, followed by a groin pressure bandage for 6 h, which was then removed. For procedures involving sheaths ≥8 French, a figure-of-eight suture was applied for additional compression at the puncture site, followed by at least 5 min of manual compression. The figure-of-eight suture was removed after 6 h, and a second groin pressure bandage was applied for an additional 6 h. No protamine was administered.

Post-intervention, a visual, palpatory, and acoustic check of the groin was conducted immediately after the procedure, one day later, and before discharge. An ultrasound examination was performed if there was an indication of an access-related complication during the clinical examination.

### 2.4. Statistics

We analyzed the variables using descriptive and inductive statistics based on their scale of measurement. Categorical variables were presented as absolute and relative frequencies, while continuous variables were expressed as either the mean ± standard deviation (SD) or the median with interquartile range, as appropriate. For categorial variables, inductive testing was conducted using the Chi-quadrat test or Fisher’s exact test and for continuous variables, Mann–Whitney U test or Student’s *t*-test, as appropriate. A *p*-value <0.05 was considered statistically significant. To determine the necessary number of patients required to demonstrate a significant effect on the primary composite endpoint, we conducted a sample size calculation based on preliminary data from our center. This calculation indicated an estimated total sample size of 1130 patients (565 patients per group). Binary logistic regression was used to evaluate the impact of various factors on groin complications. Model validity was confirmed using the Omnibus test and the Hosmer–Lemeshow goodness-of-fit statistic. Furthermore, we assessed the variables included in the regression model for multicollinearity using the variance inflation factor.

The statistical analysis and graph generation were performed using SPSS Statistics (version 29.0.1.0, IBM, Armonk, NY, USA) and Excel (version 16.75.2, Microsoft, Redmond, WA, USA).

### 2.5. Study Endpoints and Definitions

The primary endpoint was a composite of peri-interventional major and minor VAC occurrence. The secondary endpoints were major and minor VACs. Major and minor groin bleeding was defined in accordance with the recommendations of the Bleeding Academic Research Consortium (BARC) [14]. Minor vascular complications were defined as groin bleeding/hematoma < BARC 2. Major VACs were defined as groin bleeding/hematoma ≥ BARC 2, peri-interventional transfusion, pseudoaneurysm, and arteriovenous fistula.

## 3. Results

### 3.1. Study Population

A total of 1370 patients were included in this study, with 621 (45.3%) patients in the ultrasound group and 749 (54.7%) in the conventional group. Detailed patient characteristics are shown in Table 1.

### 3.2. Procedural Data

A total of 4115 punctures were performed in this study. The median number of venous groin punctures (IQR) was three (2–4) in both groups. For more details, see Table 2.

### 3.3. Complication Data

**Figure 3:**
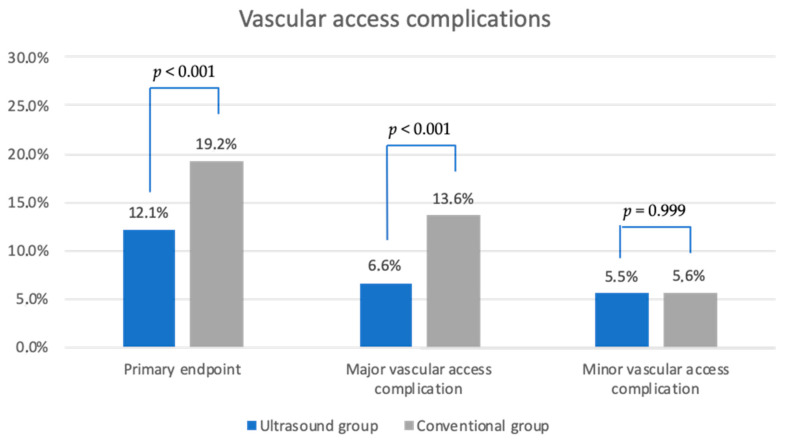
Overview of complications after venipuncture in the groin in both study groups in direct comparison.

**Figure 4:**
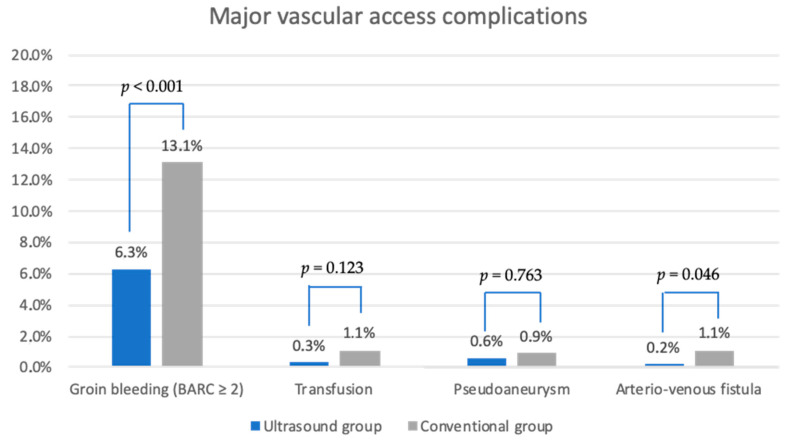
Overview of the major vascular access complications after venipuncture. BARC, Bleeding Academic Research Consortium.

The primary endpoint was achieved in 75 patients (12.1%) in the ultrasound group, compared to 144 patients (19.2%) in the conventional group (*p* < 0.001, odds ratio [95% confidence interval] 0.577 [0.427–0.781]). Major VACs were significantly fewer in the ultrasound group (*p* < 0.001, odds ratio [95% confidence interval] 0.448 [0.307–0.655]), while there was no significant difference in minor VACs between the two groups (*p* = 0.751, odds ratio [95% confidence interval] 0.975 [0.612–1.553]). For more details, see Figure 3. Figure 4 provides an overview of the components of major VACs.

The predominant proportion of major VACs was the occurrence of groin bleeding (BARC ≥ 2). The latter complication occurred significantly less in the ultrasound group (n = 39 (6.3%)) compared to the 98 patients (13.1%) in the conventional group (*p* < 0.001, odds ratio [95% confidence interval] 0.445 [0.302–0.656]). It was treated in 38 of 39 patients (97.4%) in the ultrasound group and in 93 of 98 patients (94.9%) in the conventional group by manual compression and reapplication of a groin pressure bandage solely (*p* < 0.001, odds ratio [95% confidence interval] 1.622 [1.233–2.134]). An additional figure-of-eight suture was applied in one of thirty-nine patients (2.6%) and in two of ninety-eight patients (2.0%) of the conventional group (*p* = 0.999, odds ratio [95% confidence interval] 0.602 [0.054–6.659]). Interventional repair (balloon expandable covered stent) due to arterial groin bleeding was not needed in the ultrasound group, while it was needed in three of ninety-eight patients (3.1%) in the conventional group (*p* = 0.256, odds ratio non estimable). No surgical treatment was needed.

Pseudoaneurysms were detected in four patients (0.6%) in the ultrasound group and in seven patients (0.9%) of the conventional group (*p* = 0.763, odds ratio [95% confidence interval] 0.687 [0.200–2.358]). One patient of both groups can be treated conservatively by compression therapy solely. In three ultrasound group patients (75.0%) and in six conventional group patients (85.7%), thrombin injection was performed to close the pseudoaneurysm (*p* = 0.761, odds ratio [95% confidence interval] 0.601 [0.150–2.413]).

Arterio-venous fistulas were detected in one patient (0.2%) of the ultrasound group and in eight patients (1.1%) of the conventional group (*p* = 0.046, odds ratio [95% confidence interval] 0.149 [0.019–1.198]). All fistulas could be treated conservatively in both groups.

### 3.4. Binary Logistic Regression

The analysis showed that the primary endpoint and major VACs were significantly lower in the ultrasound group than in the conventional group, even after adjusting for covariates (Table 3 and Table 4). Logistic regression indicated a 48.3% lower risk of a primary endpoint and a 55.9% lower risk of major VACs with ultrasound-guided venipuncture compared to the conventional method. Predictors of the primary endpoint and major VACs included more punctures, larger sheath diameter, therapeutic anticoagulation, and increasing age (Table 3 and Table 4). The multicollinearity assessment of the variables included in the logistic regression revealed no relevant correlation.

For minor complications, ultrasound guidance was not a predictor. Instead, factors included the international normalized ratio, partial thromboplastin time, and number of venipunctures (Table 5). All models were statistically significant and demonstrated good fit.

## 4. Discussion

This study demonstrated that ultrasound guidance for venipuncture in the groin reduces the adjusted overall risk of VACs by 48.3% and the risk of major VACs by 55.9% compared to the conventional landmark-guided puncture technique in electrophysiological procedures. Furthermore, to the best of our knowledge, this is the first study to identify that both an increased number of venous accesses and a larger sheath diameter are independent predictors of both overall and major VACs.

Depending on the definition used, access-related complications occur in 1.5% to 19.7% of patients following EP procedures [11,15,16,17]. In this study, both the overall and major VAC rates were significantly lower in the ultrasound group compared to the conventional group, with a difference that is clinically meaningful. This effect remained significant even after adjustment by logistic regression analysis.

A likely explanation is that major VACs often result from vascular injuries or unnecessary venous punctures, both of which can be minimized with ultrasound guidance. While venipuncture in the groin using a conventional landmark-guided approach relies on the presumed positional relationship between the artery and vein, significant inter-individual variability in the course of the venous vessels relative to the femoral artery is known [6]. This positional relationship can be accurately visualized in real time with ultrasound [7]. As a consequence, ultrasound guidance can help avoid unintended double-wall punctures by enabling visualization of the needle tip during the puncture [7,8]. Furthermore, ultrasound-guided puncture of the femoral veins has been shown to reduce the risk of accidental arterial punctures [9,10,11] and, thereby, the risk of treatment-required inguinal bleeding [11]. In addition to increasing the risk of bleeding, unintentional arterial punctures can cause other common complications, such as AV fistulas or pseudoaneurysms. For instance, a pseudoaneurysm can develop simply from puncturing an arterial vessel. Since ultrasound-guided groin puncture appears to reduce the occurrence of unintended arterial punctures, it is not surprising that a lower number of pseudoaneurysms was observed in the ultrasound group, although this difference did not reach statistical significance. Additionally, the significantly lower incidence of AV fistulas in the ultrasound group, along with the absence of arterial bleeding requiring intervention, could be attributed to the improved visualization of the femoral vessels provided by ultrasound guidance.

The relative reduction in overall and major VAC rates observed in this study, when comparing ultrasound-guided to conventional venipuncture, aligns with findings in the literature [1,2,7,9,11,18,19,20,21]. However, numerical comparisons of complication rates across studies reveal variability [7,9,10,11,13,18,19,20,21,22]. This variation likely stems from differences in the definitions of major VACs and patient populations. When comparing the present study with the prospective single-center study by Wynn et al., which used a similar definition of VACs, the complication rates between ultrasound-guided and landmark-guided groin puncture appear to be comparable [11]. However, when comparing these results with other studies, a numerically higher complication rate is observed in this study [7,9,18,20,22]. Wynn et al. classified bleeding that required additional compression therapy as a major VAC [11], whereas other studies did not consider this a major VAC [7,9,18,20,22]. Since over 90% of the major VACs in both groups in the present study were due to bleeding that required additional compression therapy, this likely accounts for the observed differences in overall and major VAC rates. Given that compression dressings are both immobilizing and uncomfortable for patients, and since their application involves healthcare professionals, we classified this intervention as a major VAC. Another possible reason for higher complication rates observed in this study could be the absence of protamine administration at the end of the procedure. In contrast, the literature predominantly reports the use of protamine to counteract the anticoagulants administered during the procedure, which may reduce the risk of VACs [7,9,11,13,18,20]. Additionally, it is assumed that the experience level of the interventionalists plays a crucial role in VAC outcomes, though this influence is challenging to quantify. While some studies focused exclusively on patients with atrial fibrillation, others included mixed cohorts with various electrophysiological conditions, influencing the number of groin punctures and sheath diameters used. This study demonstrates that the risk of overall and major VACs increases with the count of venous punctures and the maximum diameter of the venous sheaths. Therefore, the composition of the cohort likely contributes to the variability in complication rates reported across different studies [1]. Increasing age is associated with a heightened risk of both overall and major VACs, a finding that aligns with the existing literature [11,15]. The underlying cause of this association remains unclear.

To the best of our knowledge, this study is the first to demonstrate that the risk of access-related complications appears to increase with each venous puncture and is also influenced by the maximum sheath diameter used in the groin during EP procedures. This finding is consistent with observations by Ding et al., who reported a higher risk of complications if more than two sheaths are used per groin [13]. One possible explanation for this correlation is that each puncture is a potential source of bleeding. Additionally, a larger sheath diameter likely increases the risk of bleeding due to a larger defect in the vessel wall. Therefore, to minimize overall and major VACs during electrophysiological procedures, careful consideration should be given to sheath size and number. Interestingly, there was no significant difference in minor VACs between the ultrasound group and the conventional group. Even after adjusting for potential confounders, ultrasound guidance did not show a protective effect against minor VACs. The rates of minor complications found in this study were similar to those described in the literature [20,21,22,23]. Interestingly, other studies have also failed to demonstrate a significant difference in minor complication rates between ultrasound-guided and landmark-guided venipunctures [9,21].

The lack of a significant reduction in minor complications with ultrasound guidance may be attributed to the fact that minor bleeding is maybe not directly related to puncture technique but rather to factors affecting hemostasis. This hypothesis is supported by the logistic regression analysis, which identified an increasing number of groin punctures and a higher INR value as predictors for minor groin complications. In this context, refinements in procedural management, such as extending the duration of the figure-of-eight suture and using protamine in selected cases, particularly in fragile or high-risk patients, might help to reduce minor complications. Another important future strategy for preventing inguinal bleeding after EP procedures could be the routine use of vascular occlusion systems [24]. This approach may further support the current trend toward early or same day discharge following EP procedures [25,26].

It Is important to note that the EP procedures in this study were performed under uninterrupted oral anticoagulation, which could potentially impact the incidence of VACs, particularly bleeding events. The actual impact of peri-interventional oral anticoagulation on the incidence of bleeding events during catheter ablation procedures has not been definitively clarified [27]. In patients with atrial fibrillation, however, it is known that uninterrupted peri-interventional use of vitamin K antagonists is associated with an increased risk of major bleeding compared to direct oral anticoagulants (DOACs) [28]. In contrast, several studies have shown that a minimally peri-interventional interruption or continuous peri-interventional use of DOAKs does not appear to have a significant impact on the risk of major and minor bleeding events [12,29,30,31]. However, further prospective, multicenter studies with large numbers of patients are needed to clarify these questions conclusively.

### Limitations

This study has several limitations. First, there was a significant imbalance in the number of patients between the two groups, likely due to lower registry participation compared to the same period in the previous year. Additionally, the types of procedures performed differed between the groups, reflecting the diversity of a real-world population. This variability also accounts for differences in the number of venous and arterial groin punctures and the maximum venous sheath size. To reduce potential confounding, the results for the primary endpoint, as well as major and minor VACs, were adjusted for relevant variables. Another potential limitation of this study is the unavailability of intraprocedural ACT data in the study database.

## 5. Conclusions

In summary, ultrasound guidance for venous groin puncture in electrophysiological procedures reduces access-related complications, supporting its use with careful attention to sheath size and number.

## Figures and Tables

**Table 1:** Baseline characteristics.

	Total n = 1370	Ultrasound Group n = 621	Conventional Group n = 749	*p*-Value
Age, mean ± SD [years]	65.8 ± 14.8	65.7 ± 14.7	65.9 ± 14.9	0.871
Female, n (%) Male, n (%)	558 (40.7) 812 (59.3)	254 (40.9) 367 (59.1)	304 (40.6) 445 (59.4)	0.912
BMI, mean ± SD [kg/m^2^]	28.4 ± 6.0	28.5 ± 6.1	28.2 ± 5.8	0.291
PTT, mean ± SD [seconds]	32.9 ± 7.6	31.9 ± 7.1	33.6 ± 7.9	<0.001
INR, mean ± SD	1.17 ± 0.31	1.18 ± 0.33	1.15 ± 0.30	0.002
Platelet count, mean ± SD [Giga/L]	226.1 ± 61.9	226.4 ± 64.2	225.6 ± 59.1	0.775
Oral anticoagulation, n (%)	946 (69.1)	446 (71.8)	500 (66.8)	0.072
Vitamin-K antagonist, n (%)	36 (2.6)	17 (2.7)	19 (2.5)	
Apixaban, n (%)	388 (28.3)	174 (28.0)	214 (28.6)	
Dabigatran, n (%)	39 (2.8)	19 (3.1)	20 (2.7)	
Edoxaban, n (%)	164 (12.0)	94 (15.1)	70 (9.3)	
Rivaroxaban, n (%)	319 (23.3)	142 (22.9)	177 (23.7)	
Antiplatelet agents, n (%)	291 (21.2)	119 (19.2)	172 (23.0)	0.400
ASS, n (%)	130 (9.5)	52 (8.4)	78 (10.4)	
Clopidogrel, n (%)	136 (9.9)	58 (9.3)	78 (10.4)	
ASS + Clopidogrel, n (%)	13 (0.9)	5 (0.8)	8 (1.1)	
ASS + Ticagrelor, n (%)	1 (0.1)	1 (0.2)	0 (0.0)	
ASS + Prasugrel, n (%)	11 (0.8)	3 (0.5)	8 (1.1)	

BMI, body mass index; INR, international normalized ratio; PTT, partial thromboplastin time; SD, standard deviation.

**Table 2:** Procedural data.

	Total n = 1370	Ultrasound Group n = 621	Conventional Group n = 749	*p*-Value
Venous groin accesses				
Total per group, n	4115	1882	2235	0.377
Median per patient (IQR)	3 (2–4)	3 (2–4)	3 (2–4)	
Count of venous groin accesses				
1 access (per patient), n (%)	6 (0.6)	2 (0.3)	6 (0.8)	<0.001
2 accesses (per patient), n (%)	581 (42.4)	266 (42.8)	315 (42.1)	
3 accesses (per patient), n (%)	227 (16.6)	81 (13.0)	146 (19.5)	
4 accesses (per patient), n (%)	505 (36.9)	256 (41.2)	249 (33.2)	
5 accesses (per patient), n (%)	48 (3.5)	15 (2.4)	33 (4.4)	
6 accesses (per patient), n (%)	1 (0.1)	1 (0.2)	0 (0)	
Maximum venous sheath diameter				<0.001
5 French, n (%)	135 (9.9)	74 (11.9)	61 (8.1)	
7 French, n (%)	89 (6.5)	31 (5.0)	58 (7.7)	
8 French, n (%)	133 (9.7)	52 (8.4)	81 (10.8)	
8.5 French, n (%)	557 (40.7)	243 (39.1)	314 (41.9)	
12 French, n (%)	94 (6.9)	37 (6.0)	57 (7.6)	
12.7 French, n (%)	186 (13.6)	139 (22.4)	47 (6.3)	
13.5 French, n (%)	176 (12.8)	45 (7.2)	131 (17.5)	
Arterial groin punctures				<0.001
0 puncture (per patient), n (%)	1299 (94.8)	608 (97.9)	691 (92.2)	
1 puncture (per patient), n (%)	71 (5.2)	13 (2.1)	58 (7.7)	
Maximum arterial sheath diameter				<0.001
4 French, n (%)	56 (4.1)	11 (1.8)	45 (6.0)	
8 French, n (%)	15 (1.1)	2 (0.3)	13 (1.7)	
Type of procedure (total), n (%)	1370	621	749	
Diagnostic only	135 (9.9)	74 (11.9)	61 (8.1)	
AV-node ablation	13 (0.9)	3 (0.5)	10 (1.3)	
AVNRT	84 (6.1)	30 (4.8)	54 (7.2)	
AVRT	12 (0.9)	4 (0.6)	8 (1.0)	
CTI-dependent atrial flutter, n (%)	139 (10.1)	54 (8.7)	85 (11.3)	
Non-CTI-dependent atrial flutter, n (%)	58 (4.2)	24 (3.9)	34 (4.5)	
Focal atrial tachycardia, n (%)	50 (3.6)	21 (3.4)	29 (3.9)	
Atrial fibrillation, n (%)	786 (57.4)	387 (62.3)	399 (53.3)	<0.001
Right ventricular tachycardia, n (%)	21 (1.5)	8 (1.3)	13 (1.7)	
Left ventricular tachycardia, n (%)	72 (5.2)	16 (2.6)	56 (7.5)	

IQR, interquartile range.

**Table 3:** Logistic regression model assessing the effect of ultrasound guidance during venous groin puncture on the primary endpoint, adjusted for multiple covariates.

Parameter	Exp (B)	Confidence Interval	*p*-Value
Groin puncture under ultrasound guidance	0.517	0.372–0.719	<0.001
Age [per additional year]	1.014	1.000–1.029	0.049
Body mass index [per additional kg/m^2^]	0.989	0.962–1.016	0.411
Male sex	1.231	0.877–1.728	0.230
Number of venous groin accesses [per additional access]	1.874	1.456–2.412	<0.001
Maximum venous sheath diameter [per additional French]	1.182	1.070–1.304	<0.001
Arterial groin puncture performed	0.807	0.369–1.764	0.591
International normalized ratio [per unit]	1.266	0.751–2.133	0.376
Partial thromboplastin time [per second]	0.987	0.962–1.012	0.308
Thrombocyte count [per Giga/L]	0.998	0.995–1.001	0.180
Intake of simple platelet inhibition	0.975	0.633–1.502	0.908
Intake of dual platelet inhibition	0.949	0.254–3.541	0.938
Intake of therapeutic oral anticoagulation	2.093	1.263–3.469	0.004

Omnibus Test *p* < 0.001, Hosmer and Lemeshow Test *p* = 0.923.

**Table 4:** Logistic regression model assessing the effect of ultrasound guidance during venous groin puncture on the major complications, adjusted for multiple covariates.

Parameter	Exp (B)	Confidence Interval	*p*-Value
Groin puncture under ultrasound guidance	0.441	0.294–0.663	<0.001
Age [per additional year]	1.025	1.007–1.044	0.008
Body mass index [per additional kg/m^2^]	0.982	0.949–1.015	0.283
Male sex	1.120	0.744–1.686	0.586
Number of venous groin accesses [per additional access]	1.800	1.332–2.431	<0.001
Maximum venous sheath diameter [per additional French]	1.170	1.039–1.317	<0.001
Arterial groin puncture performed	0.954	0.390–2.333	0.918
International normalized ratio [per unit]	0.853	0.429–1.695	0.650
Partial thromboplastin time [per second]	1.007	0.981–1.034	0.592
Thrombocyte count [per Giga/L]	1.000	0.997–1.003	0.886
Intake of simple platelet inhibition	0.926	0.552–1.552	0.770
Intake of dual platelet inhibition	1.582	0.408–6.139	0.507
Intake of therapeutic oral anticoagulation	2.039	1.093–3.803	0.025

Omnibus Test *p* < 0.001, Hosmer and Lemeshow Test *p* = 0.782.

**Table 5:** Logistic regression model assessing the effect of ultrasound guidance during venous groin puncture on the minor complications, adjusted for multiple covariates.

Parameter	Exp (B)	Confidence Interval	*p*-Value
Groin puncture under ultrasound guidance	0.769	0.464–1.275	0.309
Age [per additional year]	0.996	0.975–1.017	0.692
Body mass index [per additional kg/m^2^]	1.004	0.965–1.045	0.827
Male sex	1.300	0.769–2.199	0.327
Number of venous groin accesses [per additional access]	1.752	1.173–2.616	0.006
Maximum venous sheath diameter [per additional French]	1.164	0.992–1.365	0.062
Arterial groin puncture performed	0.615	0.160–2.369	0.480
International normalized ratio [per unit]	2.296	1.125–4.686	0.022
Partial thromboplastin time [per second]	0.939	0.891–0.990	0.019
Thrombocyte count [per Giga/L]	0.995	0.991–1-000	0.055
Intake of simple platelet inhibition	1.111	0.566–2.180	0.760
Intake of dual platelet inhibition	0.000	non estimatable	0.998
Intake of therapeutic oral anticoagulation	2.084	0.950–4.570	0.067

Omnibus Test *p* = 0.018, Hosmer and Lemeshow Test *p* = 0.417.

## Data Availability

The data presented in this study are available on request from the corresponding author. The data are not publicly available due to data privacy laws.
